# Supplementary material for: Neuronal SphK1 acetylates COX2 and contributes to pathogenesis in a model of Alzheimer’s Disease
Source: Nat Commun. 2018 Apr 16;9:1479. doi: 10.1038/s41467-018-03674-2 (PMC5902554; doi:10.1038/s41467-018-03674-2)
Supplement: Supplementary file 1 — Supplementary Information(PDF 5069 kb) [file 41467_2018_3674_MOESM1_ESM.pdf]

Supplementary Information

Neuronal SphK1 acetylates COX2 and contributes to pathogenesis in a model of Alzheimer's Disease

Ju Youn Lee *et al.*

Contents

Supplementary Figure 1-18

Supplementary Tables 1-2

Supplementary Figure 1

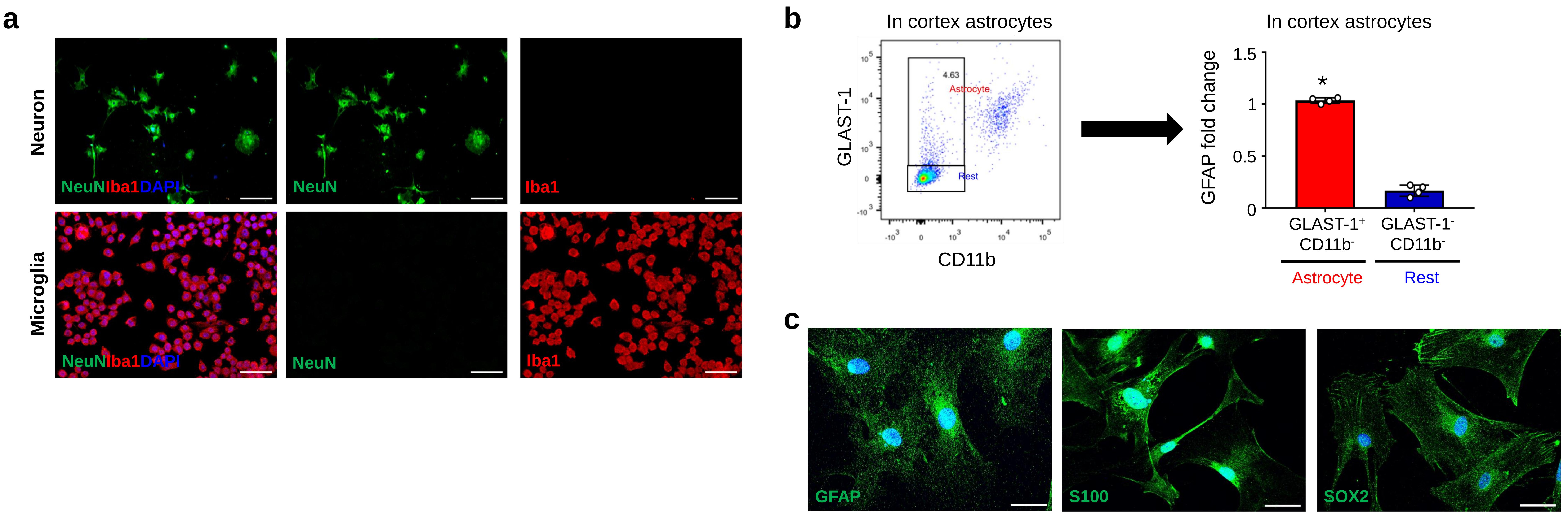

Supplementary Figure 1: Cortical neurons, microglia, and astrocytes are isolated form adult WT mice. **a,** Purity of the cortical neurons and microglia isolated from adult WT mice. Neuronal (up, NeuN, green; Scale bars, 200 μm) and microglial (down, Iba1, red; Scale bars, 50 μm) markers were detected using immunofluorescence. Nuclei were counter-stained by 4',6-diamidino-2-phenylindole (DAPI, blue). **b,** Transcript levels of *GFAP* in GLAST-1^{+}CD11b^{-} cell population and the remaining population (n = 4 per group). **c,** Immunolabeling for astrocyte markers (GFAP, S100, and SOX2). Scale bar, 50 μm. b, Student's t test, **P* < 0.05. All error bars indicate s.e.m.

Supplementary Figure 2

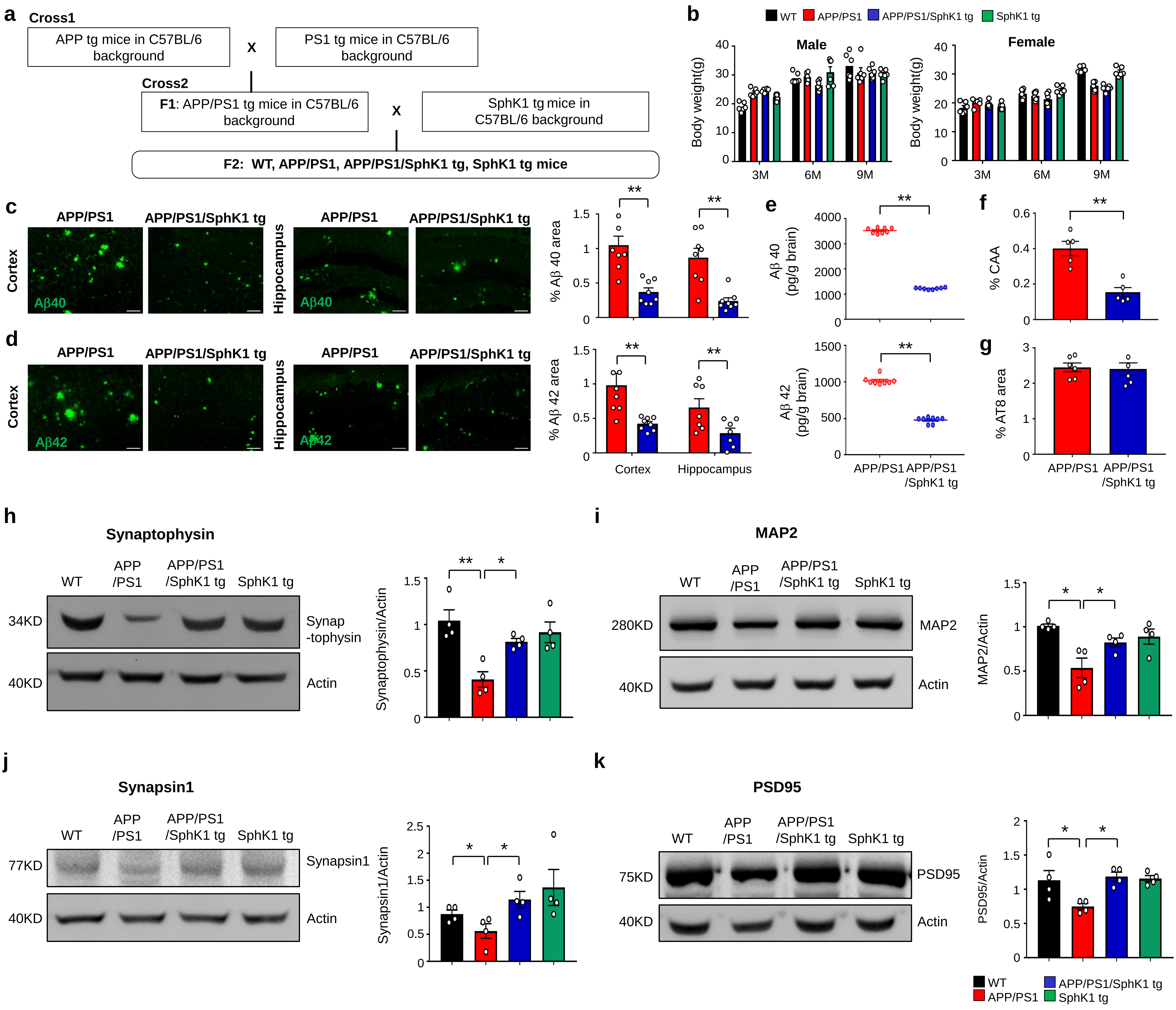

Supplementary Figure 2: Elevation of SphK1 leads to decreased AD pathology in the APP/PS1 mice. **a,** Generation of APP/PS1/SphK1 tg mice. **b,** The time course of body weights of WT, APP/PS1, APP/PS1/SphK1 tg and SphK1 tg mice (n = 6 per group). **c-e,** Analysis of Aβ40 and Aβ42 depositions from the mice brain samples using immunofluorescence staining (**c, d**; n = 7-8 per group, Scale bars, 80 μm) and ELISA kits (**e**; n = 8 per group). **f, g,** Quantification of amyloid angiopathy (**f**; n = 5 per group, CAA) and tau hyperphosphorylation (**g**; n = 6 per group, AT8). **h-k,** Western blot analysis and quantification of synaptophysin (**h**), MAP2 (**i**), synapsin1 (**j**) and PSD95 (**k**) in cortex of each group (n = 4). All data analysis was done at 9-mo-old mice except b. b and h-k, One-way ANOVA, Tukey's post hoc test. c-g, Student's t test. *P < 0.05, **P < 0.01. All error bars indicate s.e.m.

Supplementary Figure 3

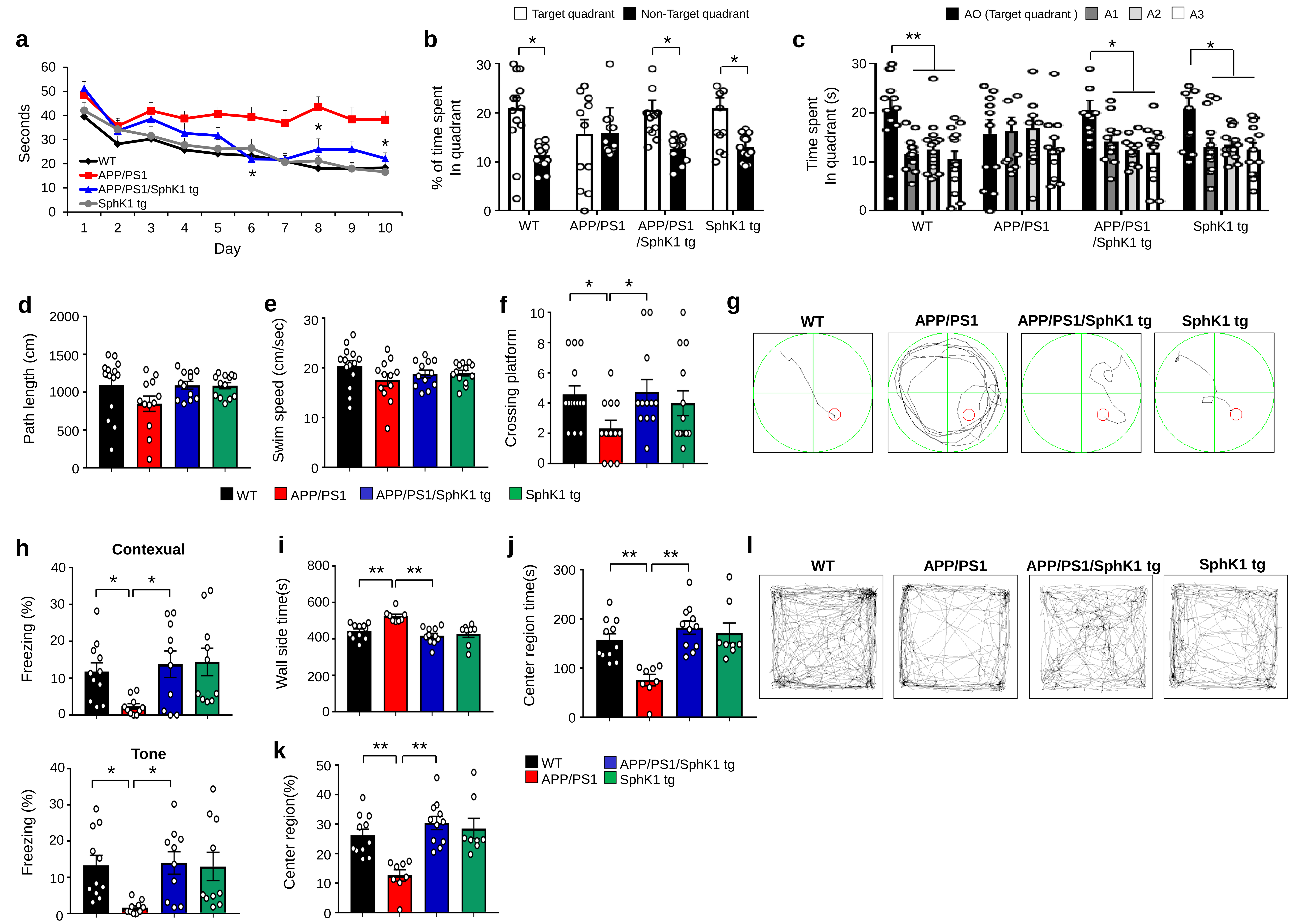

Supplementary Figure 3: Increase of SphK1 restores cognitive function in APP/PS1 mice. **a,** Learning and memory was assessed by Morris water maze test in the WT (n = 14), APP/PS1 (n = 12), APP/PS1/SphK1 tg (n = 12), and SphK1 tg (n = 13) mice. **b-e,** At probe trial day 11, time spent in target platform (**b**) and other quadrants (**c**) was measured. Path length (**d**) and swim speed (**e**) analyzed. **f,** The number of times each animal entered the small target zone during the 60 s probe trial. **g,** Representative swimming paths at day 10 of training. **h,** The results of contextual and tone tasks during fear conditioning test (n = 10-11 per group). **i, j,** Time spent in the wall side (**i**) and center regions (**j**) across during open field test (n = 8-11 per group). **k,** Percent of center region during open field test (n = 8-11 per group). **l,** Representative traces of mouse movement during open field test. All data analysis was done at 9-mo-old mice. a, c-f and h-k, One-way ANOVA, Tukey's post hoc test. b, Student's t test. *P < 0.05, **P < 0.01. All error bars indicate s.e.m.

Supplementary Figure 4

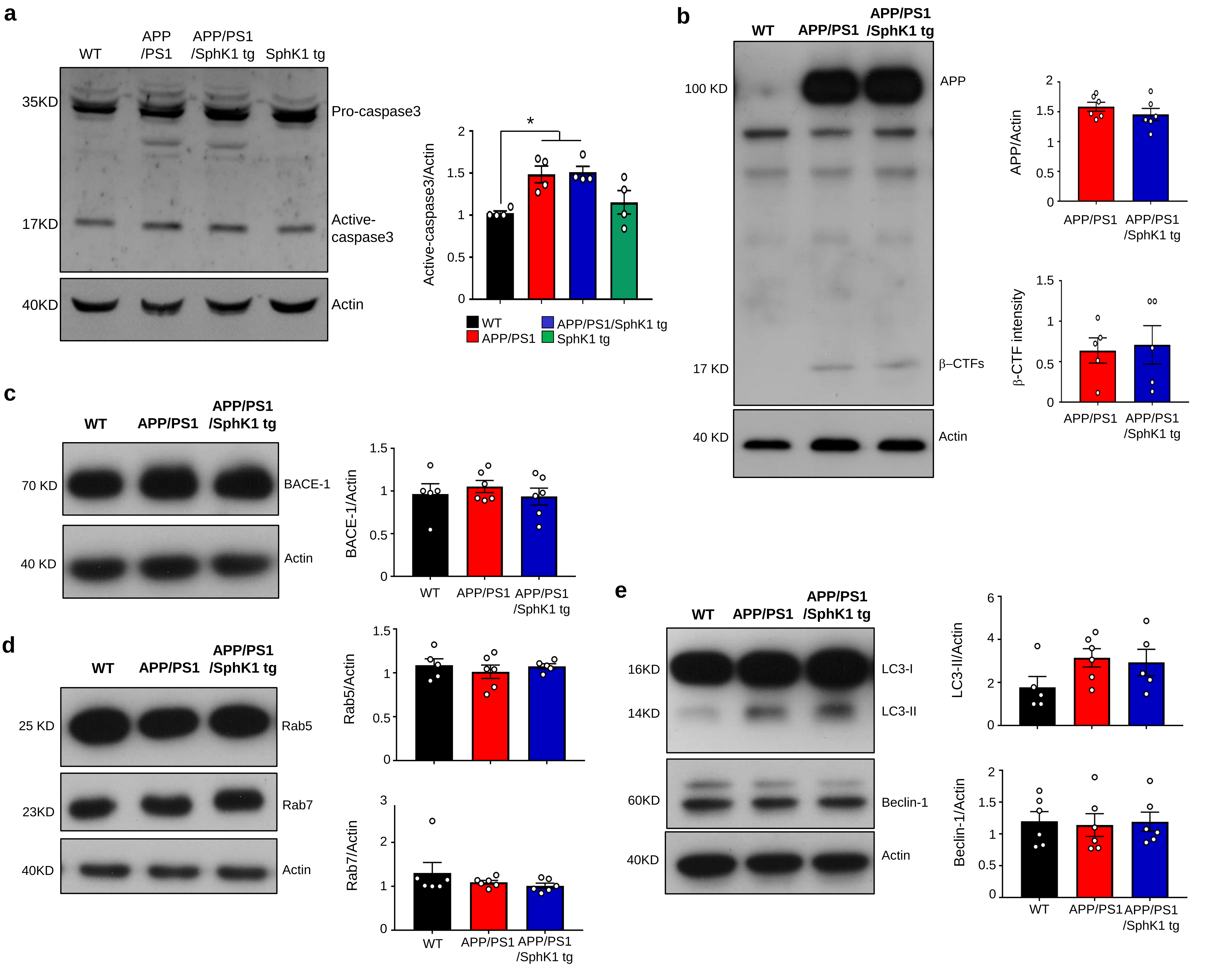

Supplementary Figure 4: Elevation of SphK1 does not affect apoptosis, processing of APP and lysosomal pathway. **a,** Western blot analysis and quantification of caspase 3 in cortex of WT, APP/PS1, APP/PS1/SphK1 tg and SphK1 tg mice (n = 4 per group). **b-e,** Western blot analysis and quantification of APP and β-CTF level (**b**), BACE-1 level (**c**), Rab5 and Rab7 (**d**), LC3 and Beclin-1 (**e**) in brain sample (n = 5-6 per group). All data analysis was done at 9-mo-old mice. a and c-e, One-way ANOVA, Tukey's post hoc test. b, Student's t test. *$P < 0.05$. All error bars indicate s.e.m.

Supplementary Figure 5

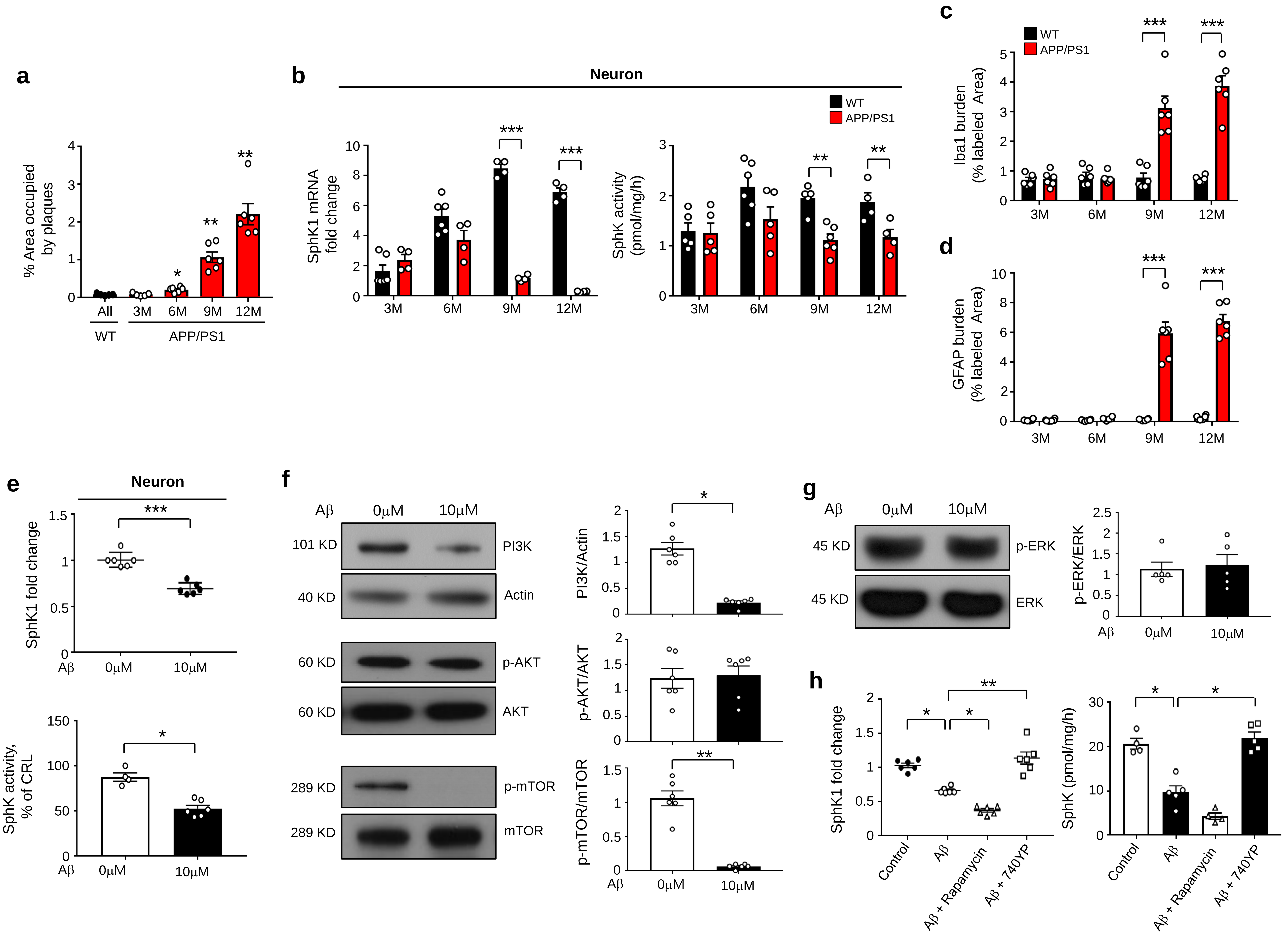

Supplementary Figure 5: Aβ reduces neuronal SphK1 through PI3K signal. **a,** The time course of quantification of thioflavin S (Aβ plaques) in the cortex of APP/PS1 mice (n = 5-6 per group). **b,** The time course of *SphK1* mRNA and SphK activity in neurons from cortex of WT and APP/PS1 mice (n = 4-6 per group). **c, d,** The time course of quantification of microglia (Iba1) (**c**) and astrocytes (GFAP) (**d**) in cortex of WT and APP/PS1 mice (n = 6 per group). **e,** *SphK1* mRNA (n = 6 per group) and SphK activity (n = 4-6 per group) in neurons after Aβ (10 μM) treatment. **f, g,** Western blot analysis for PI3K, p-AKT and p-mTOR (n = 6 per group) (**f**) and p-ERK (n = 5 per group) (**g**) in neurons after Aβ (10 μM) treatment. **h,** *SphK1* mRNA (n = 6 per group) and SphK activity (n = 4-5 per group) were assessed in Aβ treated neurons with or without rapamycin and 740YP. a and h, One-way analysis of variance, Tukey's post hoc test. b-g, Student's t test. *P < 0.05, **P < 0.01, ***P <0.001. All error bars indicate s.e.m.

Supplementary Figure 6

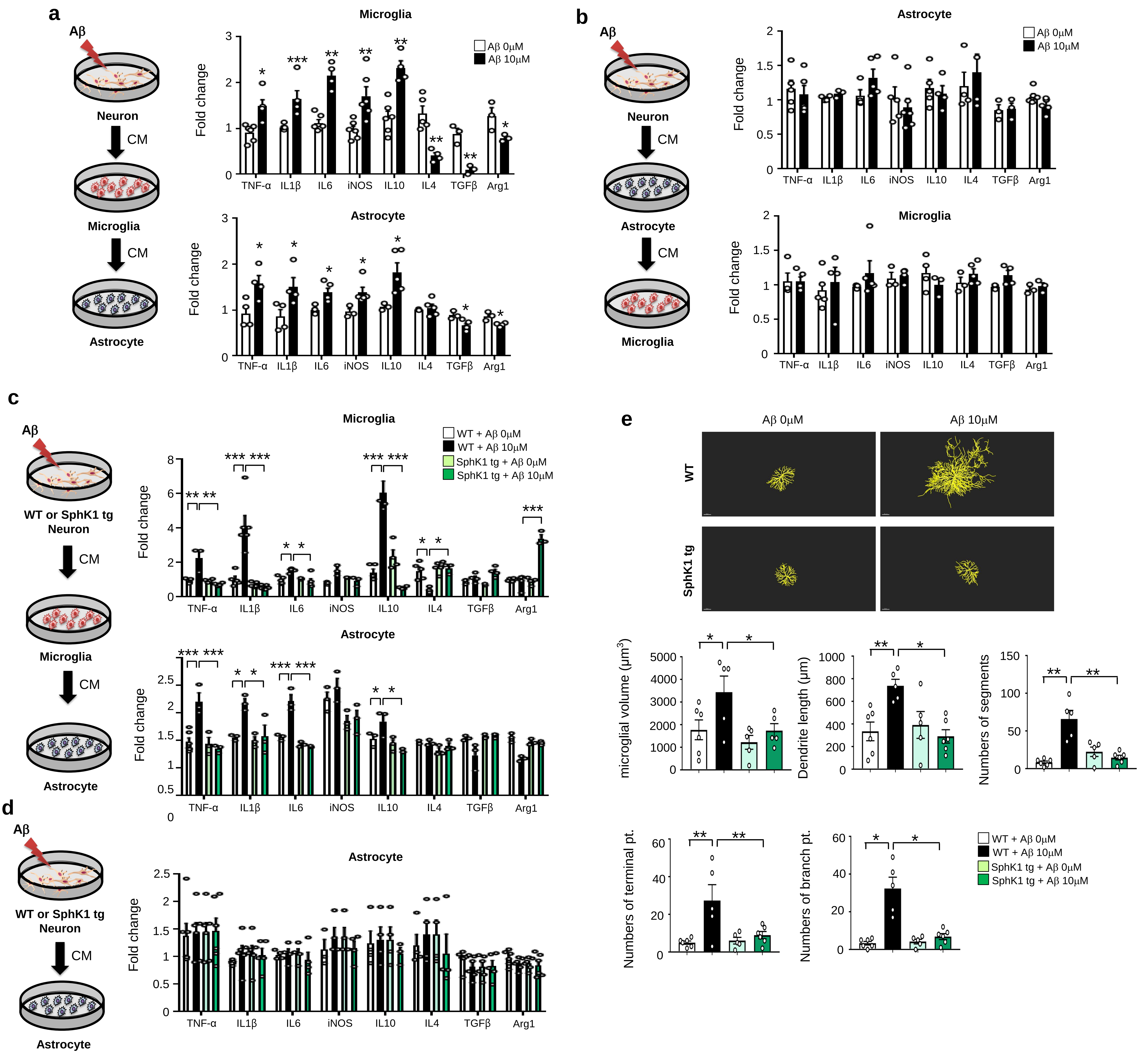

Supplementary Figure 6: Microglia are more responsive than astrocytes in SphK1-mediated inflammation by Aβ. **a,** CM was harvested from neurons treated with or without Aβ and transferred to microglia. Twenty-four hours after transfer of CM, fresh medium was added and collected on day later. This microglia medium was transferred to astrocytes, and then mRNA levels of inflammatory cytokines were analyzed in microglia (up, n = 3-6 per group) or astrocytes (down, n = 3-6 per group). **b,** CM was harvested from neurons treated with or without Aβ and transferred to astrocytes. Twenty-four hours after transfer of media, fresh medium was added and collected on day later. This astrocyte medium was transferred to microglia, and then mRNA levels of inflammatory cytokines were analyzed in astrocytes (up, n = 3-6 per group) or microglia (down, n = 3-6 per group). **c,** CM was harvested from neurons derived from WT and SphK1 tg mice with or without Aβ treatment and transferred to microglia. Twenty-four hours after transfer of media, fresh medium was added and collected on day later. This microglia medium was transferred to astrocytes, and then mRNA levels of inflammatory cytokines were analyzed in microglia (up, n = 3-6 per group) or astrocytes (down, n = 3-6 per group). **d,** CM was harvested from neurons derived from WT and SphK1 tg mice with or without Aβ treatment and transferred to astrocytes. mRNA levels of inflammatory cytokines were analyzed in astrocytes (n = 3-6 per group). **e,** Morphology of microglia treated with CM from Aβ-treated WT and SphK1 tg neurons. Up, imaris-based three-dimensional reconstruction of a representative microglia. Scale bars, 5 μm. Down, Imaris-based automated quantification of microglial morphology (n = 5-6 per group). a and b, Student's t test. c-e, One-way ANOVA, Tukey's post hoc test. *$P < 0.05$, **$P < 0.01$, ***$P < 0.001$. All error bars indicate s.e.m.

Supplementary Figure 7

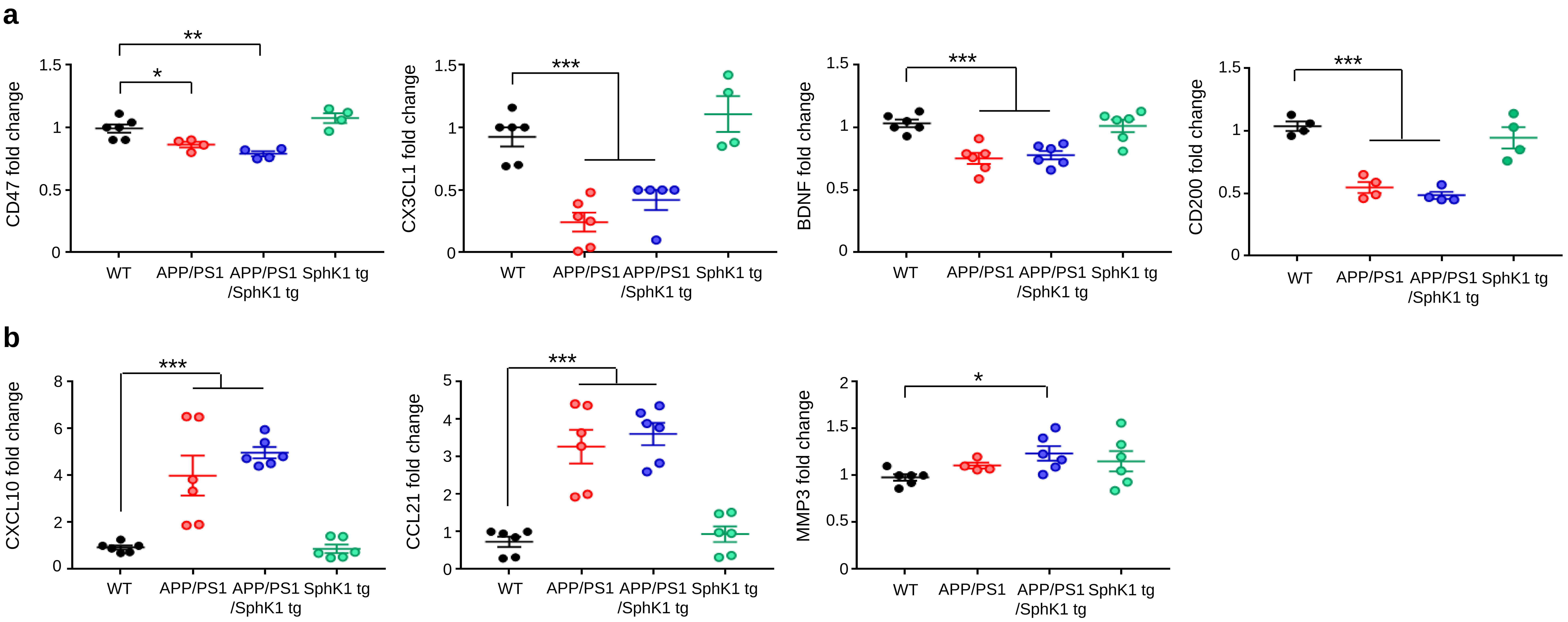

Supplementary Figure 7: Elevation of SphK1 does not affect Off/On signaling in neurons derived from APP/PS1 mice. **a,** mRNA levels of Off signal (*CD47, CX3CL1, BDNF and CD200)* in neurons derived from cortex of WT, APP/PS1, APP/PS1/SphK1 tg and SphK1 tg mice (n = 4-6 per group). **b,** mRNA levels of On signal (*CXCL10, CCL21 and MMP3*) in neurons derived from cortex of WT, APP/PS1, APP/PS1/SphK1 tg and SphK1 tg mice (n = 4-6 per group). All data analysis was done at 9-mo-old mice. a and b, One-way ANOVA, Tukey's post hoc test. **P* < 0.05, ***P* < 0.01, ****P* < 0.001. All error bars indicate s.e.m.

Supplementary Figure 8

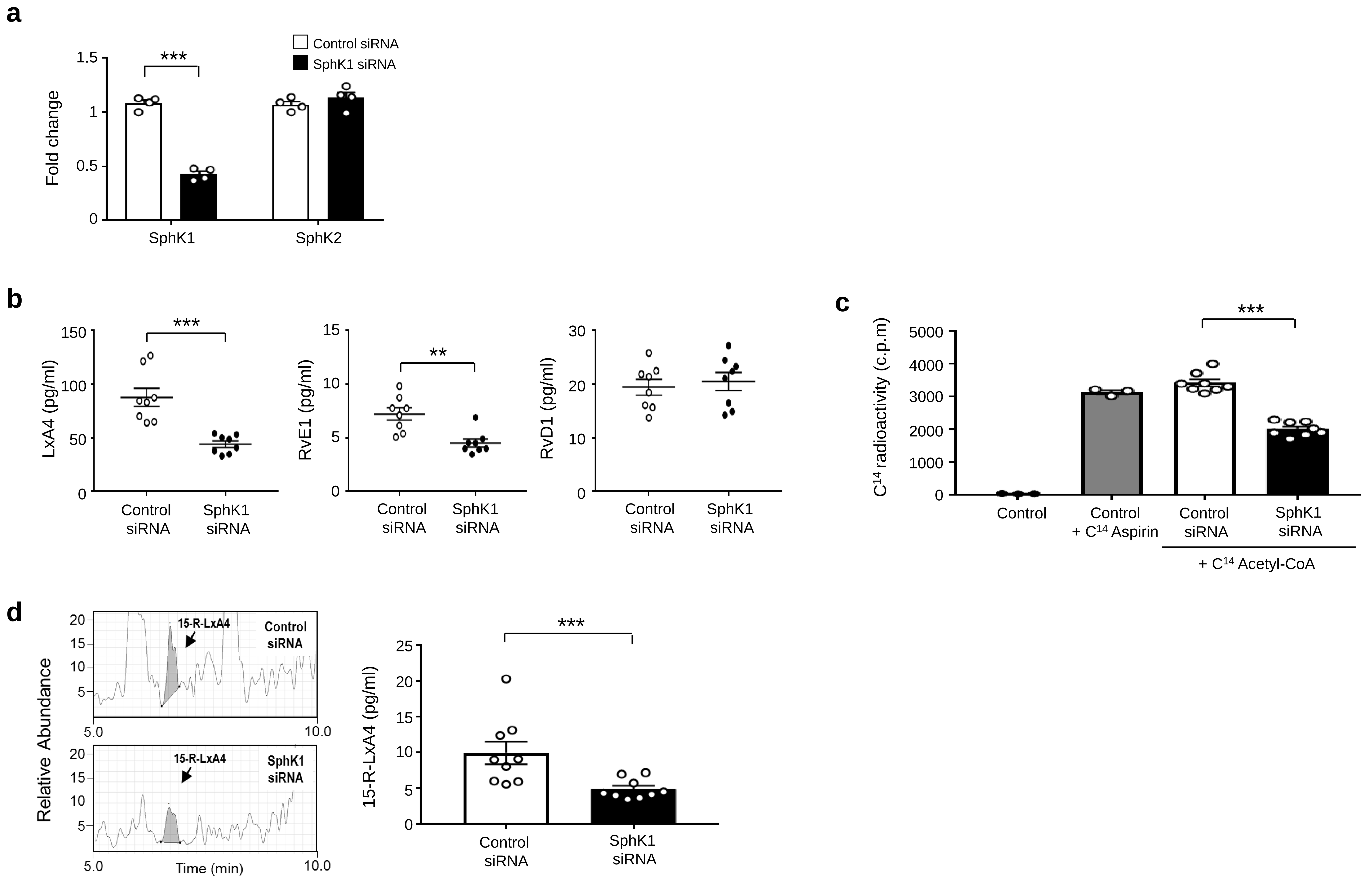

Supplementary Figure 8: Neuronal SphK1 knockdown causes reduction of COX2 acetylation and SPMs secretion. **a,** *SphK1* and *SphK2* mRNA levels in neurons treated with control siRNA and *SphK1* siRNA (n = 4 per group). **b,** Protein levels of LxA4 and RvE1 in CM of *SphK1* siRNA treated neuron, using ELISA (n = 8 per group). c, Acetylation assay of COX2 protein in *SphK1* siRNA treated neurons (n = 8 per group). d, Representative chromatograms (left panel) and quantification of 15-R-LxA4 in CM of *SphK1* siRNA treated neurons (n = 9 per group). a-d, Student's t test. ***P* < 0.01, ****P* <0.001. All error bars indicate s.e.m.

Supplementary Figure 9

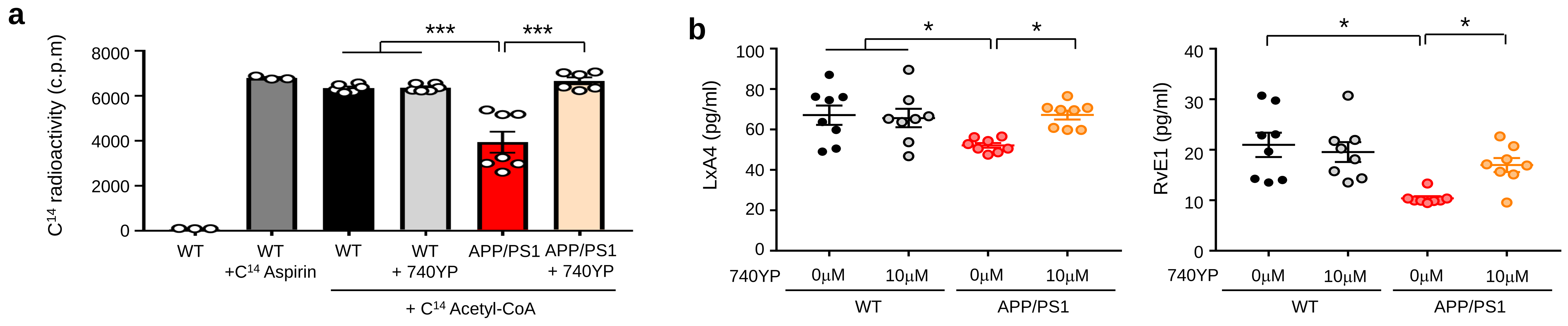

Supplementary Figure 9: Pharmacological activator of SphK1 promotes SPMs secretion by acetylating COX2 in APP/PS1 mice. **a,** Acetylation assay of COX2 protein in neurons derived from WT and APP/PS1 mice with or without 740YP treatment (2 hour after 10 μM 740YP treatment, n = 6-7 per group). **b,** Protein levels of LxA4 and RvE1 in CM of neurons derived from WT and APP/PS1 mice with or without 740YP treatment (n = 8 per group). All data analysis was done at 9-mo-old mice. a and b, One-way analysis of variance, Tukey's post hoc test. *$P < 0.05$, ***$P < 0.001$. All error bars indicate s.e.m.

Supplementary Figure 10

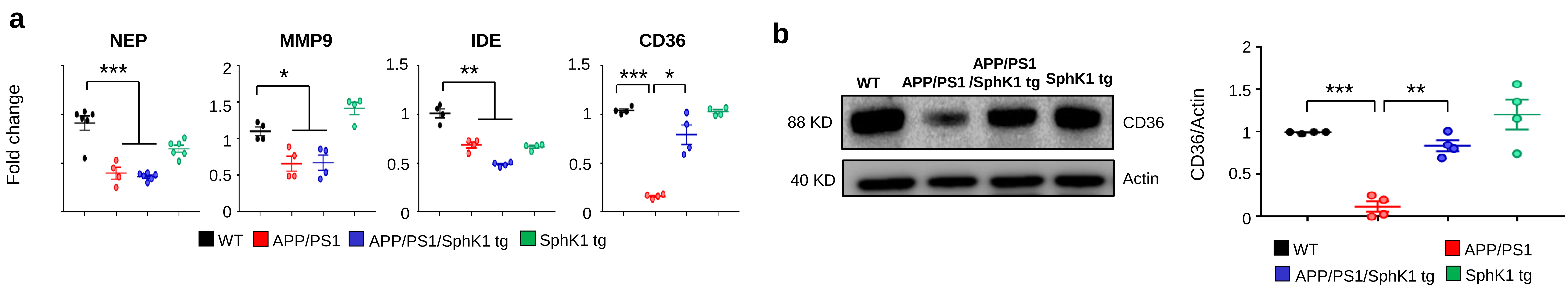

Supplementary Figure 10: Overexpression of SphK1 increase expression of CD36, but not degradation enzyme. **a,** mRNA levels of *NEP, MMP9, IDE* and *CD36* expressions in microglia derived from WT, APP/PS1, APP/PS1/SphK1 tg and SphK1 tg mice (n = 4-6 per group). **b,** Western blot analysis for CD36 in microglia derived from WT, APP/PS1, APP/PS1/SphK1 tg and SphK1 tg mice (n = 4 per group). All data analysis was done at 9-mo-old mice. a and b, One-way ANOVA, Tukey's post hoc test. **P* < 0.05, ***P* < 0.01, ****P* < 0.001. All error bars indicate s.e.m.

Supplementary Figure 11

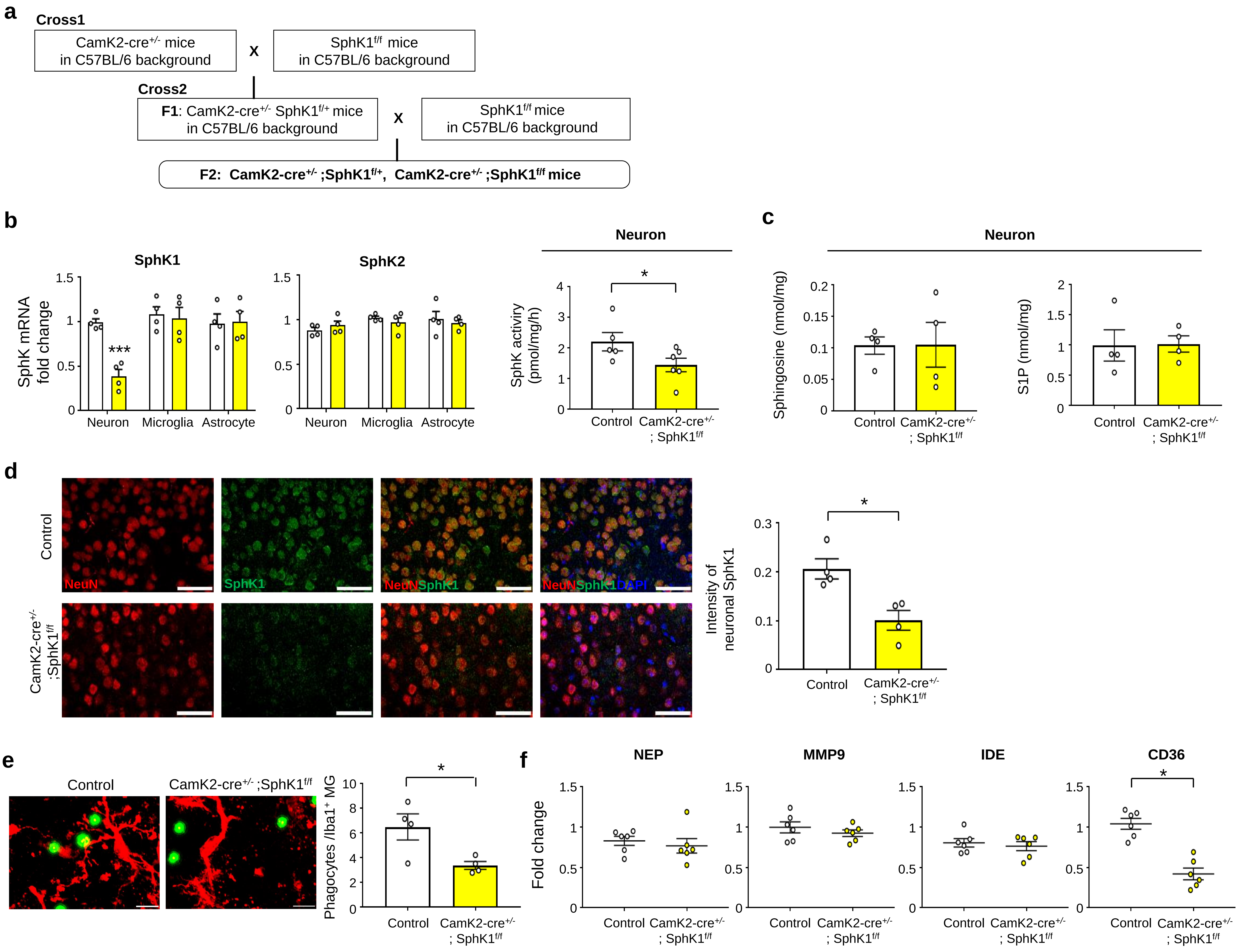

Supplementary Figure 11: Neuronal SphK1 deficiency impairs phagocytosis. **a,** Generation of CamK2-cre;SphK1 $^{flox/flox}$ mice. **b,** *SphK1* and *SphK2* mRNA (n = 4 per group) and SphK activity (n = 5-6 per group) in neurons, microglia and astrocytes derived from cortex of control and CamK2-cre;SphK1 $^{flox/flox}$ mice brain. **c,** Detection of sphingosine and S1P in neurons derived from cortex of control and CamK2-cre;SphK1 $^{flox/flox}$ mice brain (n = 4 per group). **d,** Left, representative immunofluorescence images of cortex showing SphK1 (green) merged with neuron (NeuN, red). Scale bars, 50 µm. Right, quantification of neuronal SphK1 (n = 4 per group). **e,** Left, representative photomicrograph of live slice section incubated with fluorescent beads (green). Scale bars, 10 µm. Right, quantification of the number of microglial phagocytes normalized to the total number of microglia (n = 4 per group) in brain of control and CamK2-cre;SphK1 $^{flox/flox}$ mice. **f,** mRNA levels of *NEP, MMP9, IDE* and *CD36* in microglia derived from cortex of control and CamK2-cre;SphK1 $^{flox/flox}$ mice (n = 6 per group). All data analysis was done at 2-mo-old mice. b-f, Student's t test. *P < 0.05, ***P < 0.001. All error bars indicate s.e.m.

Supplementary Figure 12

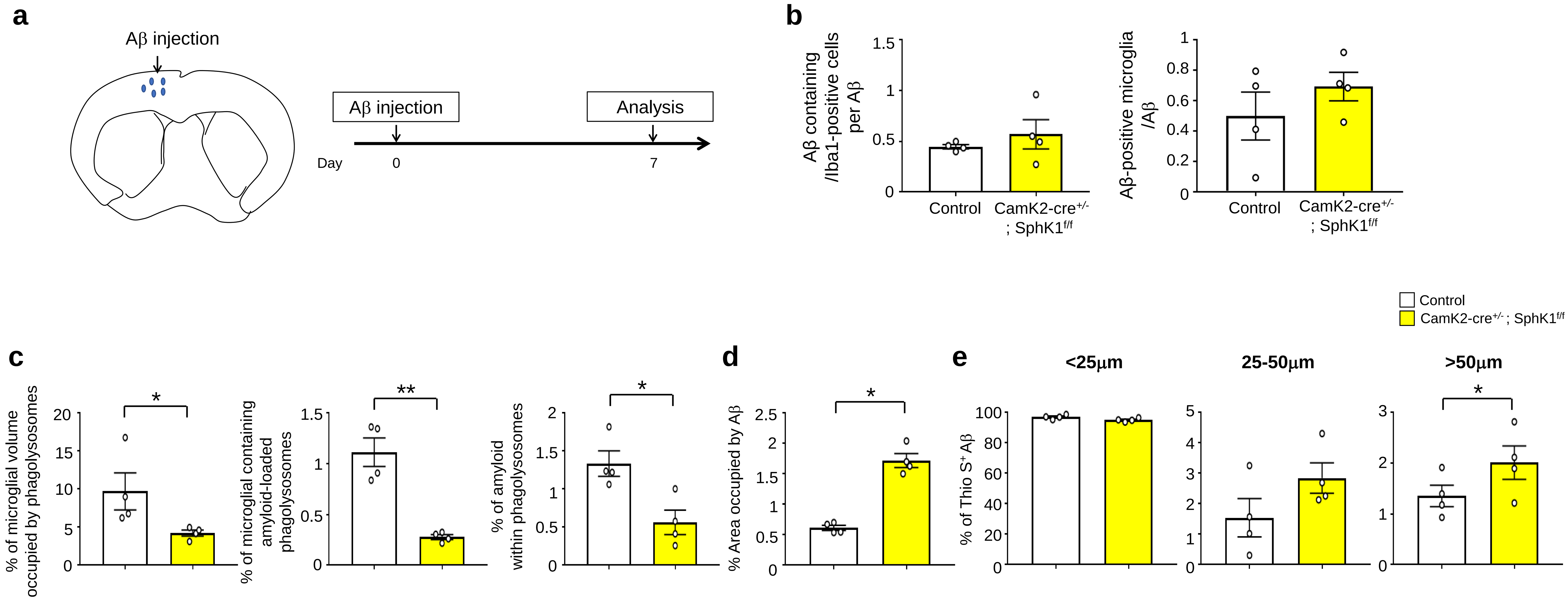

Supplementary Figure 12: Neuronal SphK1 deficiency decreases Aβ phagocytosis by microglia. **a,** Experimental schedule for Aβ injection and analysis. Aβ phagocytosis was tested in control and CamK2-cre;SphK1 $^{flox/flox}$ mice after Aβ cortical injection. **b,** Colocalization of microglia with Aβ (n = 4 per group). **c,** Quantitation of microglial volume occupied by Lamp1^{+} phagolysosomes, percent of microglia containing Aβ-loaded phagolysosomes and Aβ encapsulated in phagolysosomes (n = 4 per group). **d,** Quantitation of area occupied by Aβ in brain of control and CamK2-cre;SphK1 $^{flox/flox}$ mice after Aβ cortical injection (n = 4 per group). **e,** Morphometric analysis of Aβ (n = 4 per group). Brain sections were labeled with thioflavin S and Aβ were counted and assigned to three mutually exclusive size categories based on maximum diameter: small < 25 μm; medium 25-50 μm; or large > 50 μm. All data analysis was done at 2-mo-old mice. b-e, Student's t test. *$P < 0.05$, **$P < 0.01$. All error bars indicate s.e.m.

Supplementary Figure 13

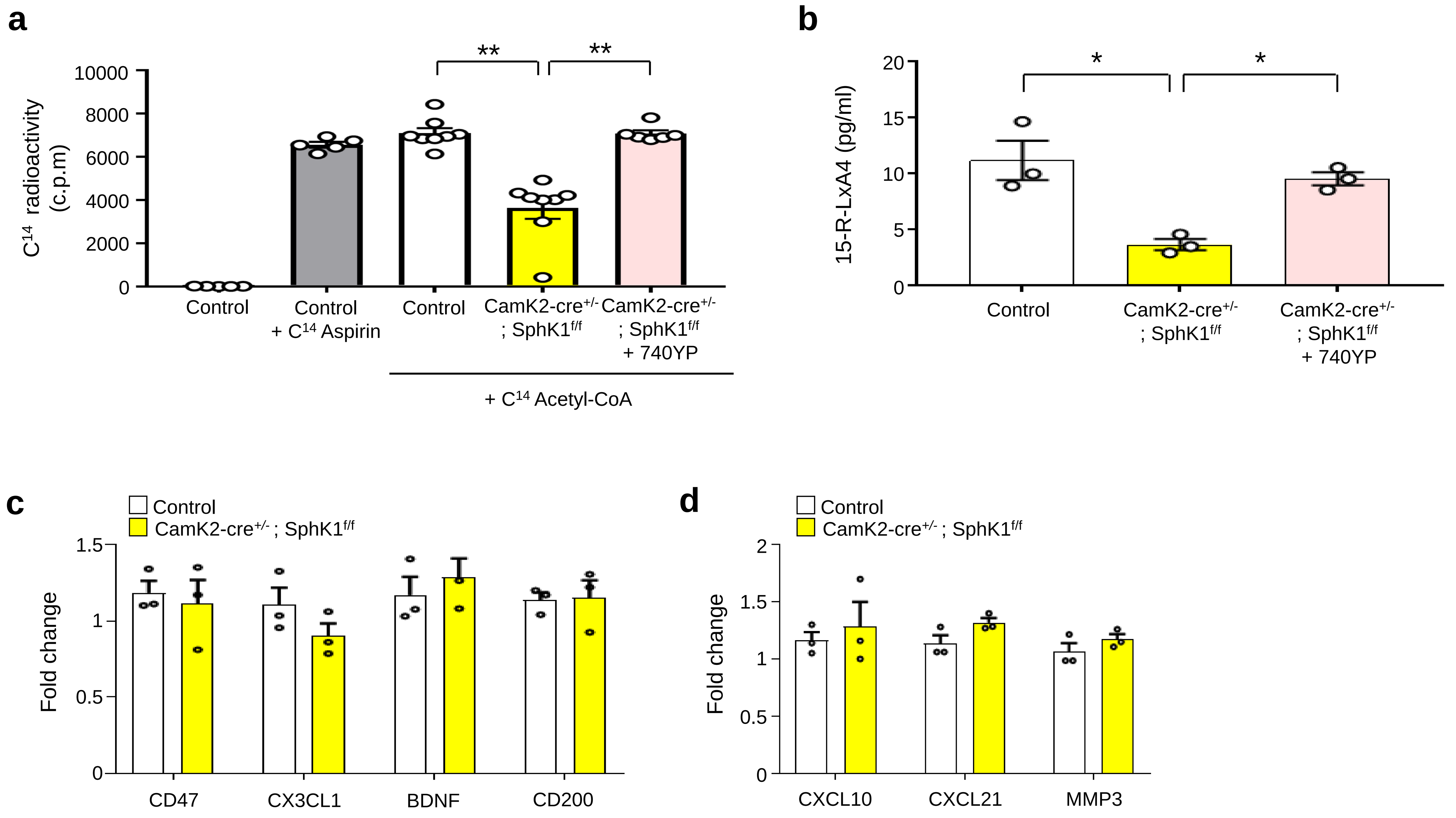

Supplementary Figure 13: Neuronal SphK1 affects acetylation of COX2 and secretion of SPM in neurons, but not Off/On signaling in CamK2-cre;SphK1 flox mice. **a,** Acetylation assay of COX2 protein in neurons derived from control and CamK2-cre;SphK1 $^{flox/flox}$ mice with or without 740YP treatment (2 h after 10 μM 740YP treatment). [^{14}C] aspirin treated neuron was positive control. Scintillation counting of acetylation assay using [^{14}C] acetyl-CoA and COX2 protein in neurons (n = 6-8 per group). **b,** Quantification of 15-R-LxA4 in neurons derived from cortex of control and CamK2-cre;SphK1$^{flox/flox}$ mice brain with or without 740YP treatment (2 h after 10 μM 740YP treatment) (n = 3 per group). **c, d,** mRNA levels of Off signal (**c**) and On signal (**d**) in neurons derived from control and CamK2-cre;SphK1 $^{flox/flox}$ mice (n = 3 per group). All data analysis was done at 2-mo-old mice. a and b, One-way ANOVA, Tukey's post hoc test. c and d, Student's t test. *P < 0.05, **P < 0.01. All error bars indicate s.e.m.

Supplementary Figure 14

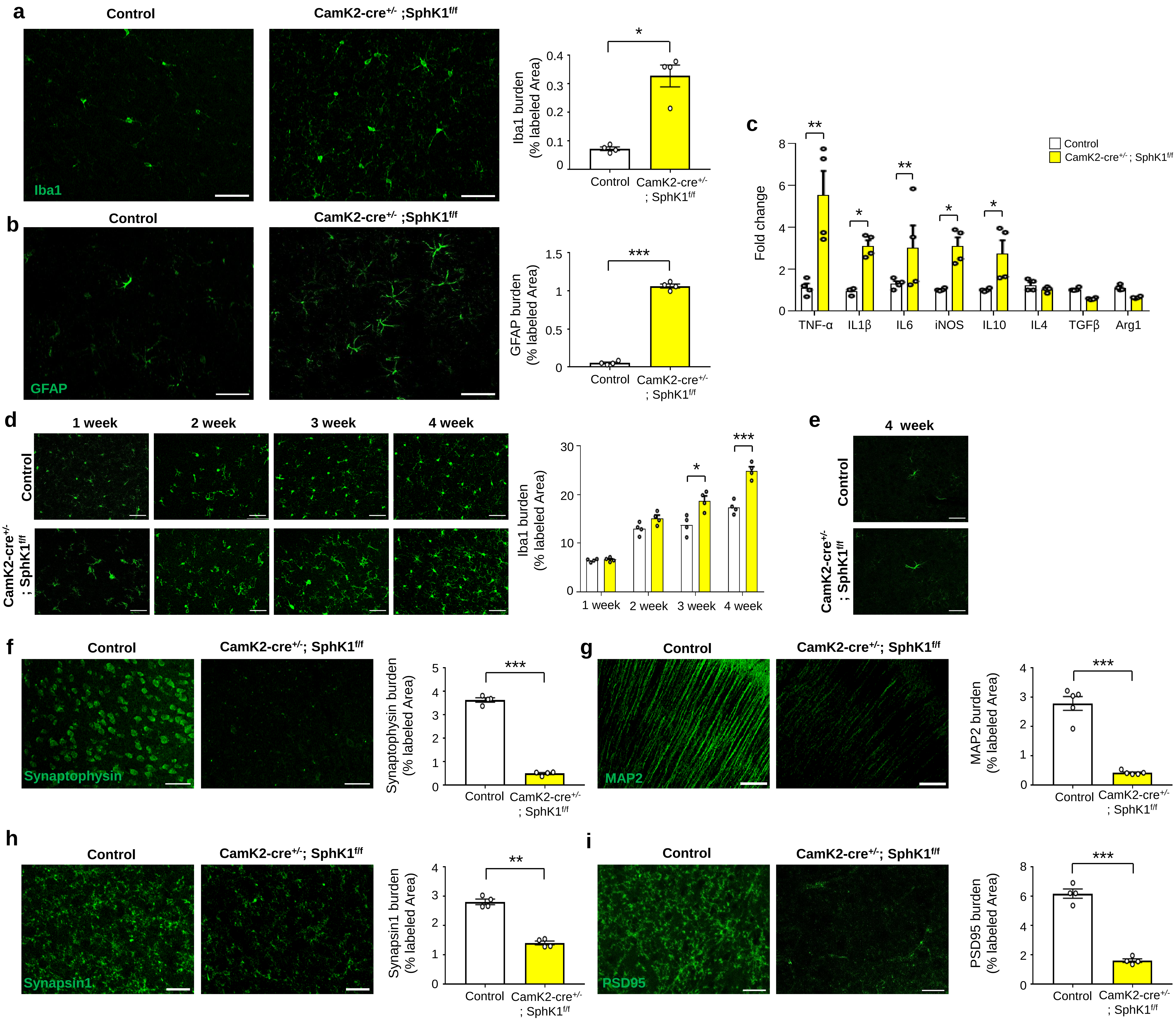

Supplementary Figure 14: Neuronal SphK1 deficiency increases neuroinflammation and decreases synaptic integrity. **a, b,** Representative immunofluorescence images and quantification of Iba1 (**a**; n = 4 per group; Scale bars, 50 μm) and GFAP (**b**; n = 4 per group; Scale bars, 50 μm). **c,** mRNA levels of inflammatory markers in cortex of control and CamK2-cre;SphK1 $^{flox/flox}$ mice (n = 4 per group). **d,** The time course of microglia quantification in cortex of control and CamK2-cre;SphK1 $^{flox/flox}$ mice (n = 4 per group; Scale bars, 50 μm). **e,** Immunofluorescence images of GFAP in cortex of control and CamK2-cre;SphK1 $^{flox/flox}$ mice at age of 4 week. Scale bars, 50 μm. **f-i,** Representative immunofluorescence images and quantification of synaptophysin (**f**; n = 4 per group; Scale bars, 50 μm), MAP2 (**g**; n = 5 per group; Scale bars, 100 μm), synapsin1 (**h**; n = 4 per group; Scale bars, 30 μm) and PSD95 (**i**; n = 4 per group; Scale bars, 30 μm) in cortex of each mice. All data analysis was done at 2-mo-old mice except d and e. a-i, Student's t test. **P* < 0.05, ***P* < 0.01, ****P* < 0.001. All error bars indicate s.e.m.

Supplementary Figure 15

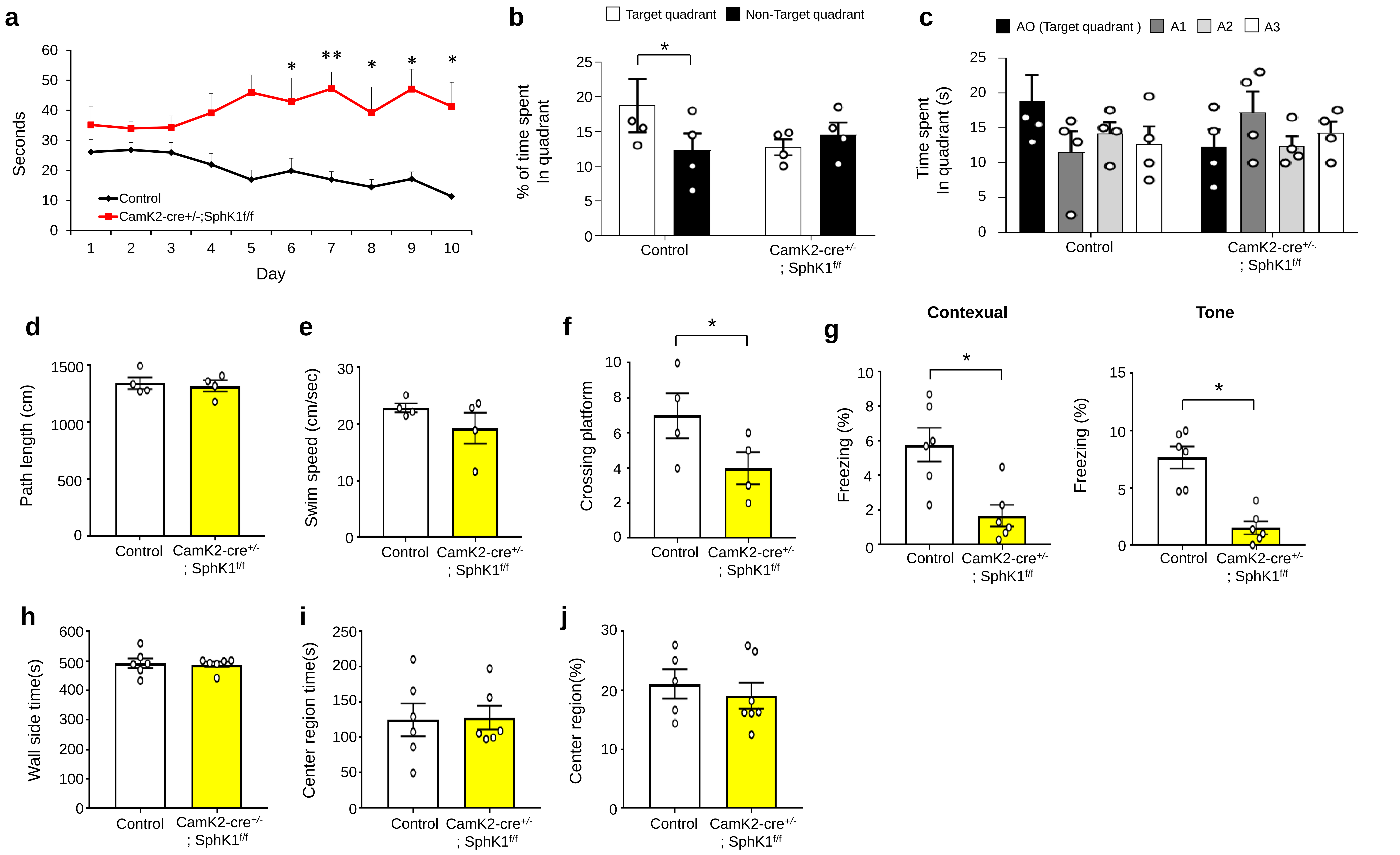

Supplementary Figure 15: Neuronal SphK1 deficiency impairs cognitive function. **a,** Learning and memory was assessed by Morris water maze test in the control and CamK2-cre;SphK1 $^{flox/flox}$ mice (n = 4 per group) **b-e,** At probe trial day 11, time spent in target platform (**b**) and other quadrants (**c**) was measured. Path length (**d**) and swim speed (**e**) analyzed. **f,** The number of times each animal entered the small target zone during the 60 s probe trial. **g,** The results of contextual and tone tasks during fear conditioning test (n = 6 per group) **h, i,** Time spent in the wall side (**h**) and center regions across (**i**) during open field test (n = 6 per group). **j,** Percent of center region during open field test (n = 6-7 per group). All data analysis was done at 3-mo-old mice. a, b and d-j, Student's t test. c, One-way ANOVA, Tukey's post hoc test. **P* < 0.05, ***P* < 0.01. All error bars indicate s.e.m.

Supplementary Figure 16

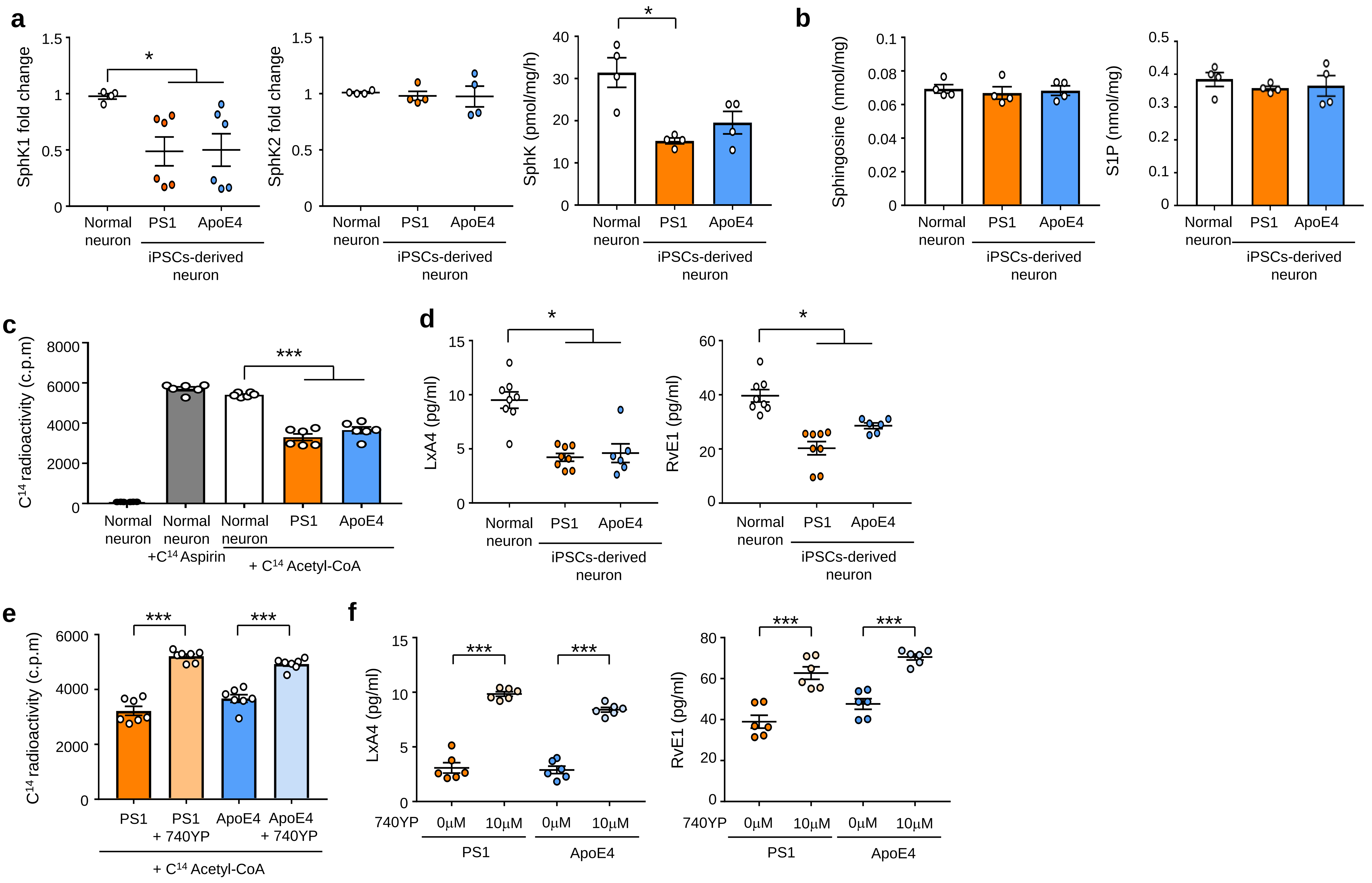

Supplementary Figure 16: SphK1 mediated COX2 acetylation and SPMs secretion are reduced in neurons derived from AD patient iPSC. **a,** *SphK1* (n = 4-6 per group) and *SphK2* mRNA (n = 4 per group) and SphK activity (n = 4 per group) in the control, PS1 and ApoE4 iPSC-derived neurons. **b,** Detection of sphingosine and S1P (n = 4 per group) in the control, PS1 and ApoE4 iPSC-derived neurons. c, Acetylation assay of COX2 protein in control, PS1 and ApoE4 iPSC-derived neurons. [^{14}C] aspirin treated neuron was positive control. Scintillation counting of acetylation assay using [^{14}C] acetyl-CoA and COX2 protein in neurons (n = 6 per group). **d,** Protein levels of LxA4 and RvE1 in CM of the control, PS1 and ApoE4 iPSC-derived neurons (n = 6-8 per group). **e,** Acetylation assay of COX2 protein in PS1 and ApoE4 iPSC-derived neurons with or without 740YP treatment (2 h after 10 μM 740YP treatment). Scintillation counting of acetylation assay using [^{14}C] acetyl-CoA and COX2 protein in neurons (n = 7 per group). **f,** Protein levels of LxA4 and RvE1 in CM derived from the PS1 and ApoE4 iPSC-derived neurons with or without 740YP treatment (n = 6 per group). a-d, One-way analysis of variance, Tukey's post hoc test. e and f, Student's t test. *P < 0.05, ***P <0.001. All error bars indicate s.e.m.

Supplementary Figure 17

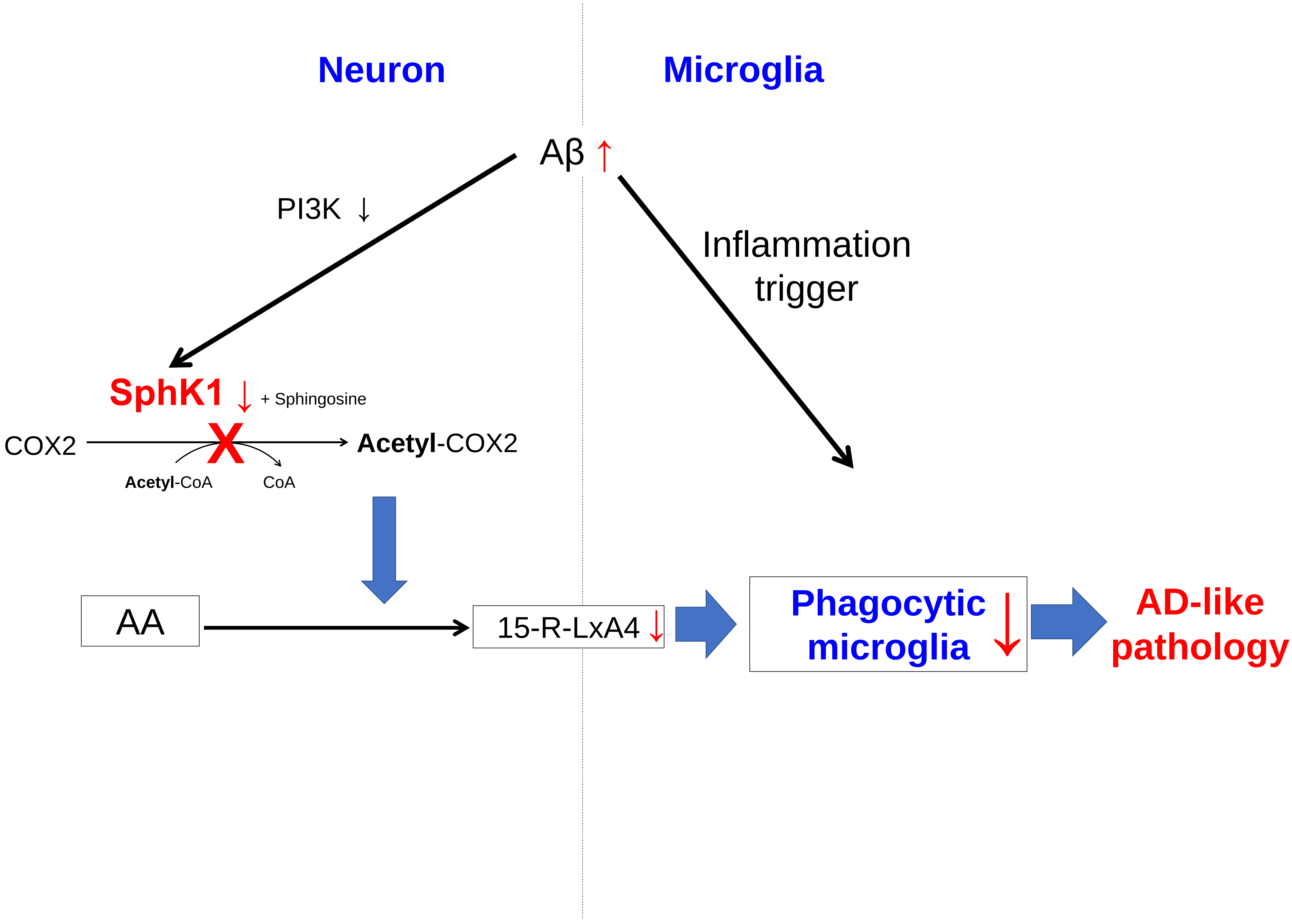

Supplementary Figure 17: Defective resolution by reduced neuronal SphK1 in AD. In AD, Aβ activates microglia and reduces neuronal SphK1 through PI3K signal. Decreased neuronal SphK1 impaired COX2 acetylation, resulting in reduction of SPMs (especially 15-R-LxA4) secretion. Decline of SPMs secretion in neuron reduces phagocytic microglia phenotype. This causes failure of resolution leading to disability of Aβ phagocytosis by microglia, persistent secretion of pro-inflammatory cytokines, and exacerbated AD-like pathology. AA, arachidonic acid.

Supplementary Figure 18

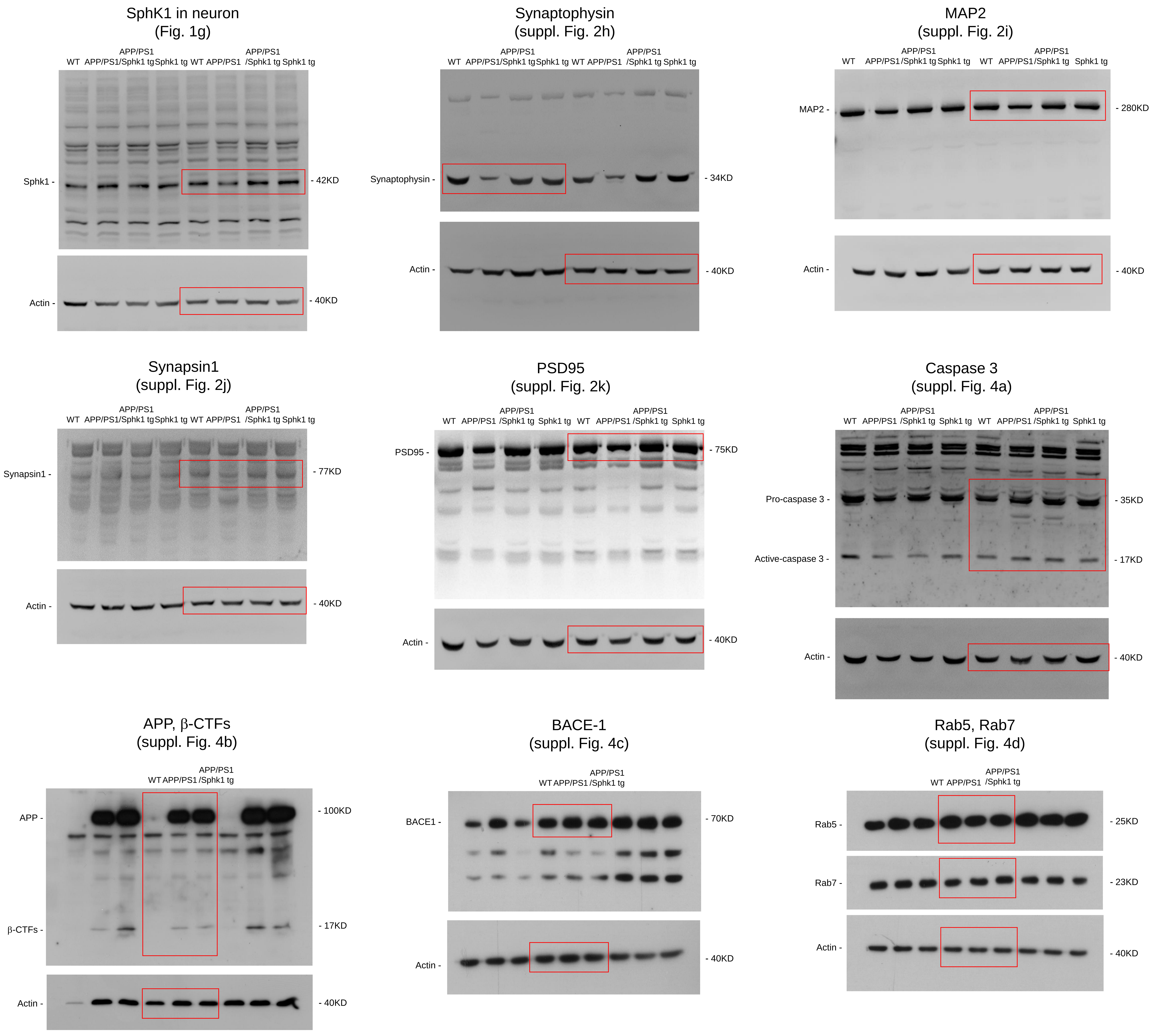

Supplementary Figure 18: Full scans of the western blots. Boxes indicate cropped images used in the figures and numbers indicate the molecular weight.

Supplementary Figure 18

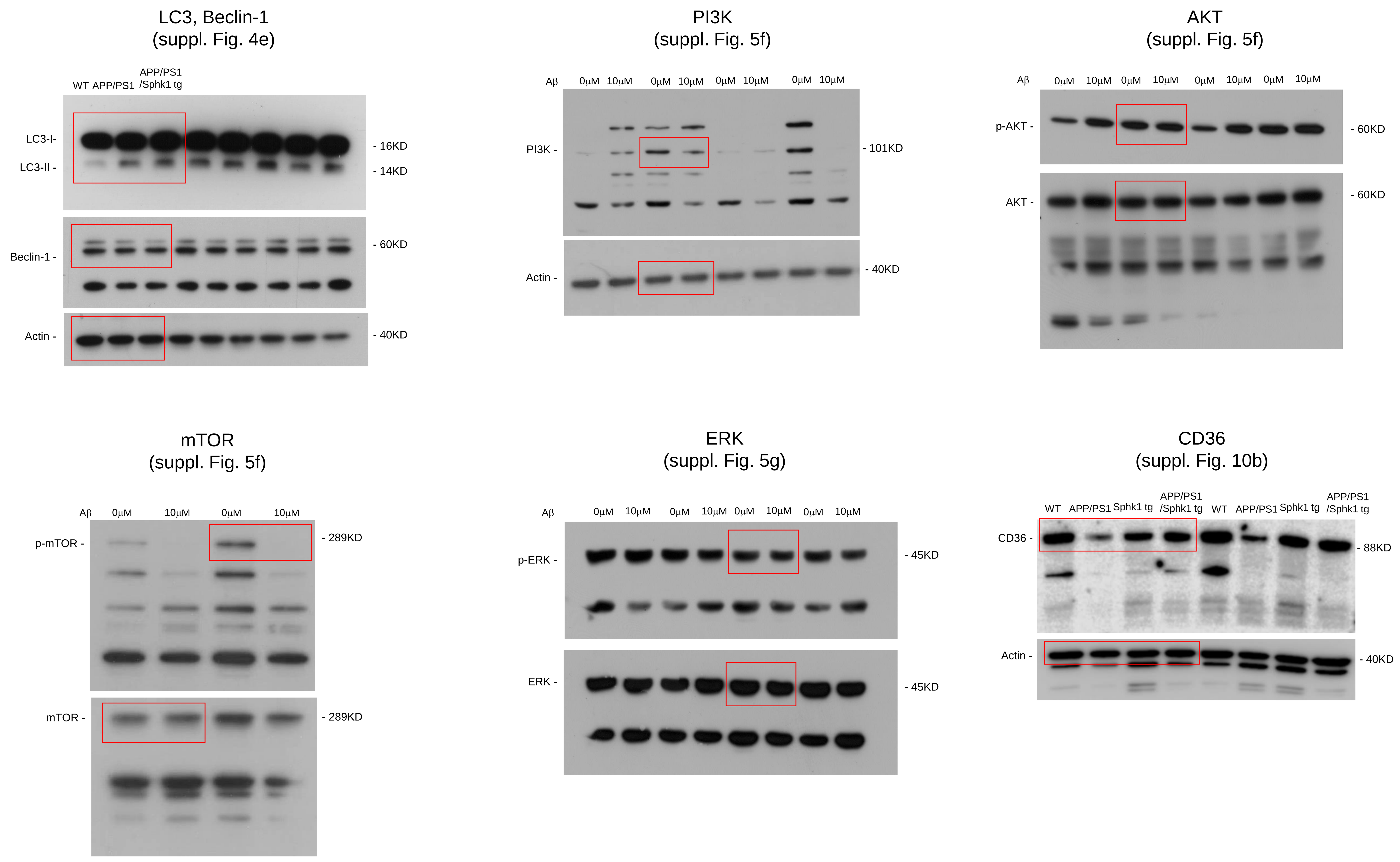

Supplementary Figure 18: Full scans of the western blots. Boxes indicate cropped images used in the figures and numbers indicate the molecular weight.

Supplementary Table

Supplementary Table 1. Human tissue information for control subjects and subjects with AD.

Case	Age	Sex	Braak stage
Normal 1	82	M	Ⅰ
Normal 2	88	M	Ⅰ
Normal 3	86	M	Ⅱ
Normal 4	87	F	Ⅱ
Normal 5	67	M	Ⅰ
Normal 6	87	F	Ⅰ
Normal 7	61	M	Ⅰ
Normal 8	78	F	Ⅰ
Normal 9	89	M	Ⅲ
Normal 10	68	M	Ⅰ
Normal 11	101	F	Ⅰ
AD 1	82	M	Ⅴ
AD 2	79	F	Ⅵ
AD 3	70	M	Ⅵ
AD 4	59	M	Ⅵ
AD 5	80	F	Ⅴ
AD 6	92	M	Ⅴ
AD 7	90	F	Ⅴ
AD 8	79	F	ⅥI
AD 9	75	M	Ⅴ
AD 10	83	M	Ⅵ
AD 11	100	M	V

Supplementary Table 2. Sequences of primer pairs.

Gene	Forward	Reverse
SphK1	5'-GGCTCTGCAGCTCTTCCAGAG-3'	5'-CTCCTCTGCACACACCAGCTC-3'
SphK2	5'-TATGTGGGAGGACCGAGTAA-3'	5'-CAAGCTCCGATCATGTCTCT-3'
GFAP	5'-CTGTCTATACGCAGCCAGGT-3'	5'-GAGGTGGAGAGGGACAACTT-3'
TNF-α	5'-GATTATGGCTCAGGGTCCAA-3'	5'-GCTCCAGTGAATTCGGAAAG-3'
IL1β	5'-CCCAAGCAATACCCAAAGAA-3'	5'-GCTTGTGCTCTGCTTGTGAG-3'
IL6	5'-CCGGAGAGGAGACTTCACAG-3'	5'-TTGCCATTGCACAACTCTTT-3'
iNOS	5'-CACCTGGAACAGCACTCTCT-3'	5'-CTTTGTGCGAAGTGTCAGTG-3'
IL10	5'-AAGGCCATGAATGAATTTGA-3'	5'-TTCGGAGAGAGGTACAAACG-3'
IL4	5'-ATCCATTTGCATGATGCTCT-3'	5'-GAGCTGCAGAGACTCTTTCG-3'
TGFβ	5'-TTACCTGGATGGAAGTGGAA-3'	5'-TGTTATGAGGAAGGGGACAA-3'
Arg1	5'-AAGCCAAGGTTAAAGCCACT-3'	5'-CGATTCACCTGAGCTTTGAT-3'
CXCL10	5'-AAAAGGGCTCCTTAACTGGA-3'	5'-GCTGGTCACCTTTCAGAAGA-3'
CCL21	5'-AGCTATGTGCAAACCCTGAG-3'	5'-CTCTTGAGGGCTGTGTCTGT-3'
MMP3	5'-GGGTAGGATGAGCACACAAC-3'	5'-TAGAAGGAGGCAGCAGAGAA-3'
CD47	5'-TGGTATCCAGCAAGCCTTAG-3'	5'-AAGACACCAGTGCCATCAAT-3'
CX3CL1	5'-CCAGAGCTGGCAATAACCTA-3'	5'-GGCATACAGGGTACGATCTG-3'
BDNF	5'-AATTTGGTAAACGGCACAAA -3'	5'-GCAAACAATCGCTTCATCTT-3'
CD200	5'-CAGGAACCCTTGATTGTGAC -3'	5'-AGTTCCAGAGTCCCAGCTCT-3'
COX2	5'-TAGAATCCAGTCCGGGTACA -3'	5'-ACAAAAGAACCCAGTGTCCA-3'
15-LOX	5'-CAGGCGTCCTCTTTGAGATA -3'	5'-GGTCTACCTGTGGTTGATCG-3'
NEP	5'-GAAATTCAGCCAAAGCAAGC-3'	5'-GATTTCGGCCTGAGGAATAA-3'
MMP9	5'-CCATGTCACTTTCCCTTCAC-3'	5'-CTCACTAGGGCAGAAACCAA-3'
IDE	5'-GAAGACAAACGGGAATACCGTG-3'	5'-CCGCTGAGGACTTGTCTGTG-3'
CD36	5'-TGTACACGGGGATTCCTTTA-3'	5'-TCCTATTGGCCAAGCTATTG -3'
GAPDH	5'-TGAATACGGCTACAGCAACA-3'	5'-AGGCCCCTCCTGTTATTATG-3'
hSPHK1	5'-GTGCAGCAAACATGCACTG-3'	5'-TGTAGGAAGAGTGGGTTCCA-3'
hSPHK2	5'-CTGACTCCTTGCTCCTACCA-3'	5'-CTCTGCTTCAAGGTGTCCAT-3'
hGAPDH	5'-TCACCCACAGGTGCCCATCT-3'	5'-GTGAGGATCTTCATGAGGTAGTCAGT-3'

SphK1, sphingosine kinase1; *SphK2*, sphingosine kinase2; *GFAP*, slial fibrillary acidic protein; *TNF-α,* tumor necrosis factor-alpha; IL1β, interleukin 1 beta; IL6, interleukin 6; *iNOS*, inducible nitric oxide synthas e; *IL10*, interleukin 10; *IL4*, interleukin 4; *TGFβ*, transforming growth factor beta; *Arg1*, arginase 1; *CXCL10*, chemokine (C-X-C Motif)ligand 10; *CCL21*, chemokine (C-C Motif) Ligand 21; *MMP3*, matrix metallopepti

dase 3; *CD47*, cluster of differentiation 47; *CX3CL1*, chemokine (C-X3-C Motif) ligand 1; *BDNF*, brain-derived neurotrophic factor; *CD200*, cluster of differentiation 200; *COX2*, cyclooxygenase 2; *15-LOX*, arachidonate 15-lipoxygenase; *NEP*, neprilysin; MMP9, matrix metallopeptidase 9; *IDE*, insulin degrading enzyme; *CD36*, cluster of differentiation 36; *GAPDH*, glyceraldehyde 3-phosphate dehydrogenase.
